# A Miniaturized Impedimetric Immunosensor for the Competitive Detection of Adrenocorticotropic Hormone

**DOI:** 10.3390/s17122836

**Published:** 2017-12-07

**Authors:** Nan Li, Egor M. Larin, Kagan Kerman

**Affiliations:** Department of Physical and Environmental Sciences, University of Toronto Scarborough, 1265 Military Trail, Toronto, ON M1C 1A4, Canada; nan.li@utoronto.ca (N.L.); egor.larin@mail.utoronto.ca (E.M.L.)

**Keywords:** electrochemical, biosensor, adrenocorticotropin hormone, impedance, screen-printed gold electrode

## Abstract

Adrenocorticotropic hormone (ACTH) plays an essential role in regulating corticosteroid hormone production, which has important functions in a myriad of critical physiological functions. In this proof-of-concept study, a miniaturized immunosensor was developed for the highly sensitive detection of ACTH using electrochemical impedance spectroscopy (EIS) in connection with disposable screen-printed gold electrodes (SPGEs). A film of 3,3′-dithiobis[sulfosuccinimidylpropionate] (DTSSP) was prepared to immobilize anti-ACTH antibodies covalently on the nanostructured SPGE surface. The surface-immobilized anti-ACTH antibodies captured the biotinylated ACTH (biotin-ACTH) and non-labelled ACTH for the competitive immunoassay. After coupling of a streptavidin-alkaline phosphatase conjugate (Streptavidin-ALP), the bio-catalysed precipitation of an insoluble and insulating product onto the sensing interface changed the charge transfer resistance (R_ct_) characteristics significantly. The detection limit of 100 fg/mL was determined for ACTH in a 5 μL sample volume, which indicated that this versatile platform can be easily adapted for miniaturized electrochemical immunosensing of cancer marker biomolecules. High selectivity and sensitivity of our immunoassay to detect ACTH in real samples demonstrated its promising potential for future development and applications using clinical samples.

## 1. Introduction

Adrenocorticotropic hormone (ACTH) is a 39-amino acid peptide hormone (4.5 kDa) released from the anterior pituitary gland [1]. ACTH is an essential component of the hypothalamic-pituitary adrenal axis, regulating corticosteroid hormone production, which has important functions in a myriad of critical physiological functions [2,3,4]. Dysregulation of ACTH secretion, resulting from conditions such as hypopituitarism [5] or Cushing’s syndrome [6], can be life threatening if not diagnosed and treated properly, and in the most severe cases, it can cause death because of vascular collapse [7]. ACTH-secreting pancreatic neuroendocrine tumours, although rare, were reported to be responsible for about 15% of ectopic Cushing syndrome [8,9]. They represent a challenging entity because their diagnosis is frequently difficult, and clear-cut morphologic criteria useful to differentiate them from other types of symptoms have not been described [8,9]. For early and accurate assessment of altered ACTH secretion, advances in its detection are required; however, there are challenges associated with the diagnosis for altered ACTH level. ACTH deficiency is often misdiagnosed due to the general symptoms such as weight loss, vomiting, nausea, and muscle weakness. Furthermore, the fluctuation of ACTH in serum (<4.1 to 51.4 pg/mL) makes diagnosis even more problematic [10]. To address these challenges in diagnosis, a rapid, sensitive, and selective detection method is needed. Various detection methods exist for the detection of ACTH, such as radioimmunoassays [11,12], chemiluminescence assays [13], and enzyme-linked immunosorbent assays [14]. Current issues concerning these methods include sensitivity and specificity along with expensive bench-top instrumentation that requires skilled technicians and time-consuming procedures [15]. In this report, we present a proof-of-principle study on impedimetric detection of ACTH using disposable screen-printed gold electrodes (SPGEs). Our immunoassay is a cost-effective approach that requires low sample volumes. SPGEs can be mass-produced at low-cost, and each experiment can be performed on a new and analogous surface to prevent possible cross-contamination errors between surfaces that were exposed to biological samples [16]. Each SPGE can be discarded after use and eliminate carry-over contamination from tedious cleaning, and require less reagents for detection [17,18,19]. Furthermore, current advances in instrumentation allow these SPGEs to be compatible with portable devices that are in the size of a smart-phone for convenient point-of-care measurements [20,21,22,23].

## 2. Materials and Methods

### 2.1. Reagents

Anti-ACTH polyclonal antibody was purchased from EMD Millipore (Darmstadt, Germany). Biotin-labelled adrenocorticotropic hormone (ACTH) (1–39, Human) and non-labelled ACTH (1–39, Human) were purchased from AnaSpec (Fremont, CA, USA). Streptavidin-conjugated alkaline phosphatase (Streptavidin-ALP), disodium 5-bromo-4-chloro-3-indolyl phosphate (BCIP), 3,3′-dithiobis[sulfosuccinimidylpropionate] (DTSSP) and ethanolamine were purchased from Sigma-Aldrich (Oakville, ON, Canada). The screen-printed gold electrodes (SPGEs) were obtained from BioDevice Technology (Ishikawa, Japan). Follicle-stimulating hormone (FSH), human growth hormone (hGH), rat blood, and plasma samples were kindly prepared and donated by Dr. Paul Le Tissier (University of Edinburgh, Edinburgh, UK). Other reagents were of analytical grade, and were used as received.

### 2.2. Surface Modification and Electrochemical Impedance Spectroscopy

**Figure 1:**
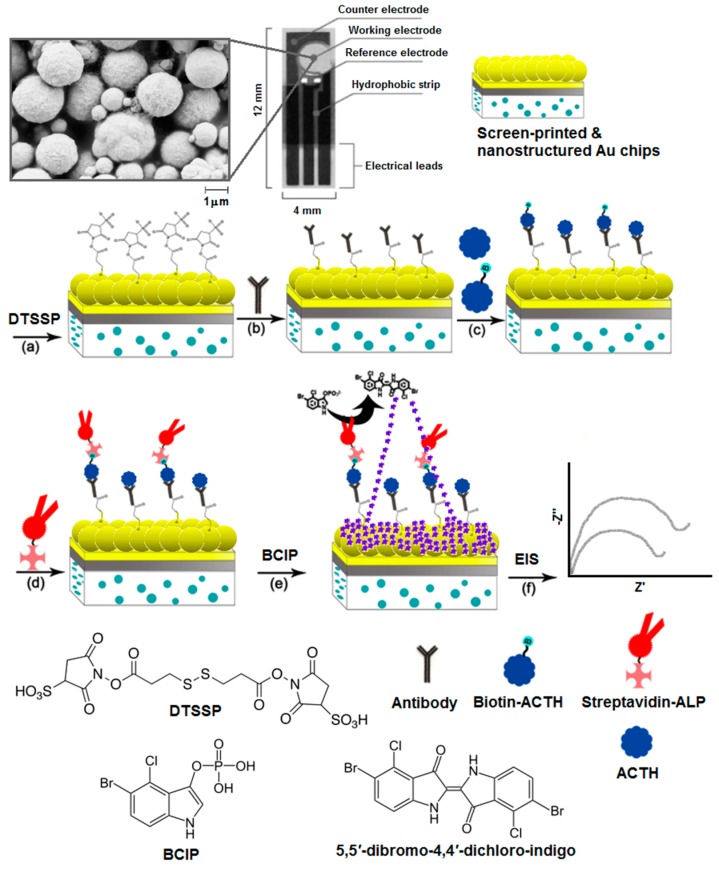
Illustration for the SPGE-based detection of ACTH. (**a**) Self-assembled film of 3,3′-dithiobis[sulfosuccinimidylpropionate] (DTSSP) was formed on nanostructured gold surface. (**b**) NHS-moieties of DTSSP enabled the covalent immobilization of antibodies on the surface. (**c**) The biotinylated adrenocorticotropic hormone (biotin-ACTH) was captured by immobilized antibodies. (**d**) Streptavidin-conjugated alkaline phosphatase (Streptavidin-ALP) was then coupled to biotin-ACTH. (**e**) Amplification reaction was initiated by adding the water-soluble substrate mixture, 5-bromo-4-chloro-3-indolyl phosphate (BCIP), which produced insoluble indigo dimer (see inset for dimerization reaction). (**f**) Electrode interface was monitored using electrochemical impedance spectroscopy (EIS) after each surface modification step.

**Figure 2:**
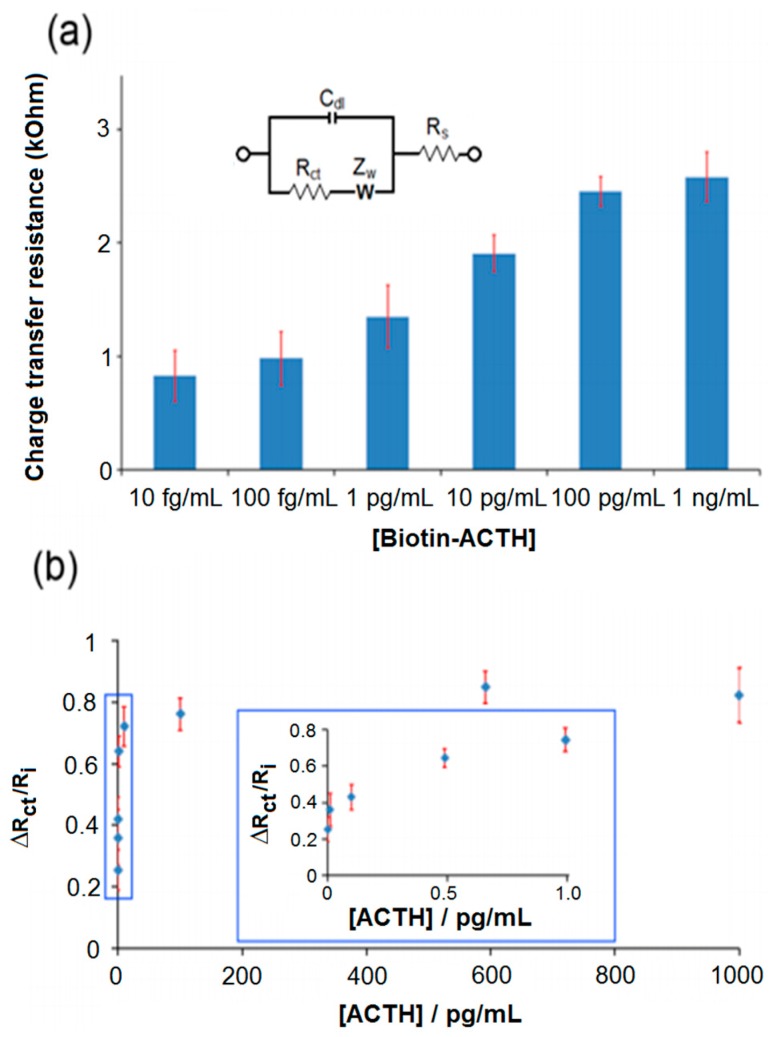
(**a**) Plot for the R_ct_ of various biotin-ACTH concentrations after fitting the raw data to equivalent circuit model using CNLS regression. (**b**) Plot for the R_ct_ ratio of various non-labelled ACTH concentrations after fitting the raw data to equivalent circuit model using CNLS regression Error bars indicate the standard deviation of six replicate measurements (*n* = 6). Other conditions were as described in the Section 2.

The principal of the detection method is shown in Figure 1. (a) A layer of 3,3′-dithiobis[sulfosuccinimidylpropionate] (DTSSP) was formed on SPGE surface through Au–S bonding. The working electrode of the SPGE was incubated with 80 μL of 2 mM DTSSP in 100 mM Na_2_CO_3_ for 48 h at −4 °C. DTSSP acted as the linker molecule to immobilize the antibodies covalently on the nanostructured surface. The preparation of nanostructures with scanning electron microscope images were described in our previous publication [21]. An aliquot (15 μL) of anti-ACTH antibodies at a desired concentration in 50 mM phosphate buffer solution with 100 mM NaCl (pH 7.4) was spotted onto the working electrode surface and incubated for 12 h at −4 °C. (c) An aliquot (5 μL) of the desired concentration of biotin-ACTH was incubated on the surface for 30 min. The antibodies interacted with the biotin-labelled ACTH (biotin-ACTH). The antibody-modified electrodes were thoroughly rinsed with water and immersed in 100 mM ethanolamine solution (ethanol) for 1 h at 25 °C in order to block all unreacted NHS active ester groups. (d) An aliquot (20 μL) of streptavidin-conjugated alkaline phosphatase (Streptavidin-ALP) was spotted on the electrode surface and incubated for 1 h. Biotin moieties captured the streptavidin-ALP. (e) Finally, an aliquot (20 μL) of 0.3 mM disodium 5-bromo-4-chloro-3-indolyl phosphate (BCIP) was spotted on the surface and incubated for 10 min to form the insoluble 5,5′-dibromo-4,4′-dichloro indigo product. As the product precipitated, it formed an insulating layer on the surface [24,25,26,27]. (f) For the competitive detection of ACTH, known concentrations of non-labelled ACTH were mixed with 100 pg/mL biotin-ACTH and exposed to the antibody-modified SPGEs followed by the same experimental steps as described above. As non-biotinylated ACTH from spiked samples displaced the biotinylated ACTH (not shown in this image), streptavidin-ALP conjugates were also rinsed away from the surface, decreasing the formation of insoluble product. After each modification step, SPGEs were rinsed with Milli-Q water rigorously. The changes in charge transfer resistance (R_ct_) were measured using electrochemical impedance spectroscopy (EIS). EIS was performed using a μAutolab II Electrochemical Analyzer (Metrohm, Herisau, Switzerland) in conjunction with Frequency Response Analyzer (FRA) software. EIS were recorded using 10 mM [Fe(CN)_6_]^3−/4−^ as the redox probe in 50 mM PBS with 100 mM NaCl (pH 7.4) in a frequency, ω, ranging from 1 Hz to 10 kHz at a dc potential of 0.30 V, corresponding to the recorded Fe(CN)_6_^3−/4−^ standard reduction potential, with a superimposed root mean squared AC voltage amplitude of 5 mV. Analysis of the raw impedance data was based on complex non-linear least-squares (CNLS) regression fitting to the Randles equivalent circuit, as shown in Figure 2. EIS is a highly sensitive detection method and allows for a wide range of biosensing applications [28,29,30,31,32]. EIS measurements are interpreted using the Randles equivalent circuit, consisting of ohmic resistance (R_s_) of electrolyte, double layer capacitance (C_dl_), charge-transfer resistance (R_ct_), and Warburg impedance (Z_w_) [33,34,35]. The observation of Z_w_ as a component of the equivalent circuit was attributed to the nanostructured topology of the surface [23], which might have facilitated the diffusion process [36]. Upon the adsorption of insoluble indigo product on the electrode surface, the accessibility of the solution-based redox probe [Fe(CN)]^3−/4−^ to the surface was suppressed, which significantly increased R_ct_, enabling highly sensitive detection of ACTH.

### 2.3. Scanning Electron Microscopy (SEM)

SPGE surfaces were observed using a Hitachi S530 scanning electron microscope (Hitachi, Tokyo, Japan) in the Centre for the Neurobiology of Stress at UTSC. The surfaces were electrically connected to the sample stub by smearing silver paste between the SPGE and the metallic stub. The surfaces were monitored at an acceleration voltage of 20 kV with a working distance of 5.0 mm.

## 3. Results and Discussion

The electrochemical detection relied on the formation of the insoluble product on the electrode surface, which significantly increased the R_ct_ over a short period of time. Calibration studies were performed using varying concentrations of antibody and streptavidin-ALP (data not shown). The optimum concentrations for antibodies and streptavidin-ALP were determined as 10% (*v*/*v*) and 100 ng/mL, respectively. As shown in Figure 2a, concentrations between 10 fg/mL to 1 ng/mL of biotin-ACTH were calibrated. The concentration dependence of R_ct_ was studied using various concentrations of non-labelled ACTH in the presence of 1 ng/mL biotin-ACTH. The binding affinity of non-labelled ACTH was found to be stronger than that of biotin-labelled ACTH, because polyclonal antibodies were utilized in this study. A significant decrease was observed in R_ct_ after exposure of non-labelled ACTH to the polyclonal antibodies, because the non-labelled ACTH was not able to capture streptavidin-ALP due to its lack of biotin moieties.

As non-labelled ACTH displaced the biotin-ACTH on the antibody-modified surfaces, the difference between R_ct_ in the presence and absence of non-labelled ACTH increased significantly. As shown in Figure 2b, the R_ct_ ratio ΔR_ct_/Ri was calculated using the formula ΔR_ct_ = R_f_ − R_i_, where R_f_ displays the R_ct_ that was detected after exposure of antibody-modified SPGEs to non-labelled ACTH, and R_i_ displays the R_ct_ that was detected in the absence of target proteins with the rest of the experimental steps performed as described in the Materials & Methods section. The R_ct_ began to plateau beyond 100 pg/mL ACTH, suggesting a dynamic range between 100 fg/mL and 100 pg/mL in PBS. This dynamic range is 10^2^ magnitude larger than the dynamic range of the ACTH sensor published [37], offering a more versatile detection platform. A detection limit was observed as 100 fg/mL. The limit of blank was the highest analyte concentration expected to be determined, when replicates of a blank sample containing no analyte were tested. Assuming a Gaussian distribution of the raw analytical data from blank samples, the limit of blanks would represent 95% of the observed values. Thus, the limit of detection was calculated as the sum of the limit of the blank and 1.645 (95% confidence interval) of the standard deviation of the signals obtained from the lowest concentration of analyte. This detection limit was about 100-fold more sensitive than a commercially available ELISA-based kit, which had a detection limit in the 10 pg/mL range [38]. Furthermore, the selectivity of our immunosensor was challenged in the presence of interfering proteins.

**Figure 3:**
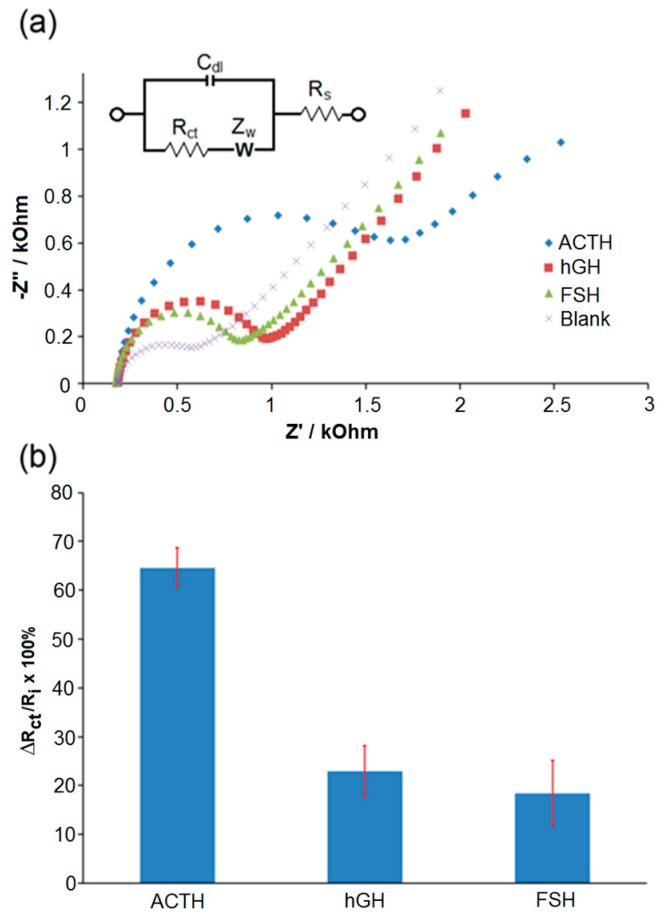
(**a**) Nyquist plots for the detection of ACTH, hGH, and FSH at 100 fg/mL in PBS fitted with the Randles equivalent circuit. (**b**) Plot for the R_ct_ ratios calculated for ACTH, hGH, and FSH after fitting the raw data to equivalent circuit model using CNLS regression. Error bars indicate the standard deviation of six replicate measurements (*n* = 6). Other conditions were as described in Section 2.

Two pituitary gland hormones, human growth hormone (hGH) and follicle stimulating hormone (FSH), that might be present in real samples were used in control experiments as shown Figure 3a. The R_ct_ percentage ΔR_ct_/R_i_ × 100% (Figure 3b) was calculated using the formula ΔR_ct_ = R_f_ − R_i_, where R_f_ displays the R_ct_ that was detected after exposure of antibody-modified SPGEs to ACTH, hGH, or FSH as the target protein in the competitive assay, and blank is the signal obtained after all modifications that were made in the absence of biotin-ACTH. R_i_ displays the R_ct_ that was detected in the absence of target proteins with the rest of the experimental steps performed as described in the Materials & Methods section. The ΔR_ct_/R_i_ percentage was significantly high for ACTH at 64% in comparison with the non-target proteins hGH and FSH. Relatively low ΔR_ct_/R_i_ ratios of 23% and 18% for hGh and FSH, respectively, were attributed to much lower non-specific adsorption of those proteins on the electrode surface. Control experiments were performed to further challenge the immunosensor using various plasma and whole blood samples.

**Figure 4:**
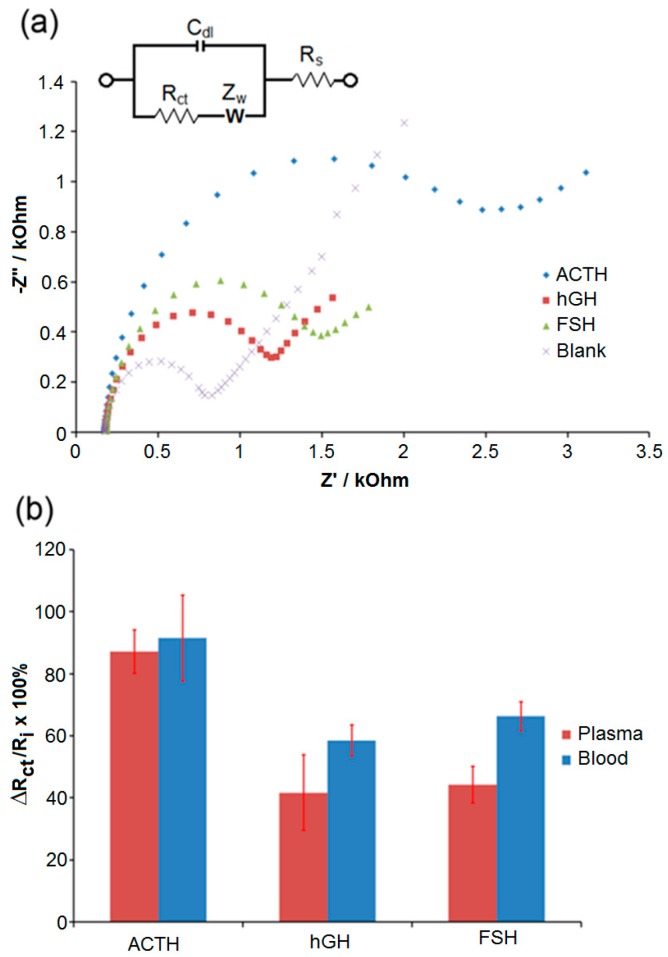
(**a**) Representative Nyquist plots for the detection of ACTH, hGH, and FSH in undiluted plasma as fitted with the Randles equivalent circuit. (**b**) Plot for the R_ct_ ratios calculated for ACTH, hGH, and FSH in whole blood (red) and plasma (blue) samples after fitting the raw data to equivalent circuit model using CNLS regression. Error bars indicate the standard deviation of six replicate measurements (*n* = 6). Other conditions were as described in Section 2.

After 100 fg/mL ACTH, hGH, and FSH were spiked in undiluted plasma (Figure 4a and Figure 4b-blue bars) and blood (Figure 4b-red bars), those samples were used as the target protein solutions in the experimental steps as described above, blank is the signal obtained after all modifications that were made in the absence of biotin-ACTH. There was an increase in the responses, which were attributed to the fouling of surfaces after exposure to undiluted plasma and blood samples. However, ΔR_ct_/R_i_ percentage was significantly higher for ACTH in both undiluted plasma and blood samples compared to those obtained with hGH and FSH. In this study, ethanolamine was found sufficient to quench the active-NHS ester groups on the electrode surface to suppress the covalent attachment of ACTH molecules non-specifically. Bovine serum albumin (BSA) and polyethylene glycol (PEG) modifications are commonly utilized to avoid non-specific adsorption issues on electrode surfaces [35,39,40]. We have also investigated the applications of various PEGylated linker molecules and BSA on our biosensor surfaces to suppress non-specific adsorption issues, and the results of those studies will be published elsewhere.

## 4. Conclusions

Our proof-of-concept study demonstrates that electrochemical immunosensors provide a promising platform to detect rare cancer biomarkers such as ACTH in undiluted real samples. Enzymatic amplification of impedimetric response at disposable miniaturized SPGEs enabled a low detection limit. High selectivity and sensitivity of our immunoassay exemplify its promising potential for future development and applications using clinical samples.
